# Feasibility and Acceptability of a Remote Sleep–Dependent Memory Assessment in Older Adults With Cognitive Concerns: Pilot Cross-Sectional Study

**DOI:** 10.2196/87926

**Published:** 2026-06-11

**Authors:** Clara Cher Yi Tan, Aaron Lam, David Ireland, Dana Bradford, Nathan Cross, Melvern Ted Kurniawan, Simone Simonetti, Jurgen Fripp, Sharon L Naismith

**Affiliations:** 1 Healthy Brain Ageing Program, The Brain and Mind Centre, The University of Sydney, 100 Mallett Street, Camperdown, NSW, 2050, Australia, 61 293519993; 2 School of Psychology, The University of Sydney, Camperdown, NSW, Australia; 3 Woolcock Institute of Medical Research, Macquarie University, Macquarie Park, NSW, Australia; 4 Australian eHealth Research Centre, Commonwealth Scientific and Industrial Research Organisation, Herston, Australia

**Keywords:** sleep-dependent memory consolidation, digital cognitive assessment, aging, cognition, sleep

## Abstract

**Background:**

Sleep-dependent memory consolidation (SDMC), the process by which sleep supports the transfer of memories into long-term storage, declines with age but remains underexplored in older adults with subjective cognitive decline and mild cognitive impairment. Traditional SDMC assessments are typically conducted in lab settings, with limited evidence for feasibility to do these assessments at home for this clinical population.

**Objective:**

To address this, we co-designed the *Sleep Memories* app, which assesses overnight memory for a previously validated 32-item word-pairs task. This pilot study first explored the feasibility and acceptability of the app, including willingness to participate. Second, we explored various demographic, clinical, and subjective sleep factors associated with task completion and SDMC performance in a memory clinic sample.

**Methods:**

Within an 8-month period, we invited 141 older adults aged 50 years and above (mean 71.27, SD 7.51 y) from the Healthy Brain Ageing clinic, a specialist brain health and memory clinic in Sydney, to pilot the *Sleep Memories* app. Of these, 76 (mean 70.19, SD 7.75) agreed to participate. All participants underwent a full neuropsychological test battery, medical assessment, and mood assessment.

**Results:**

Sixty-eight participants completed at least 1 test of the word-pairs task. The word-pairs task completion rate for all trials was over 50%. There was 57% (39/68) completion of both evening and morning delayed recall tests. Lower willingness to participate was associated with lower global cognitive scores, poorer sleep behavior, and clinical factors. Higher task completion was associated with greater education and greater anxiety levels. User feedback indicated that the app was well-accepted and liked, although some participants reported minor technical difficulties.

**Conclusions:**

These findings support the feasibility of mobile app–based SDMC assessment in older adults at risk of cognitive decline and underscore the importance of considering individual characteristics (eg, subjective sleep factors, clinical characteristics, and education) when designing digital SDMC tools.

## Introduction

Sleep is a fundamental biological process that is essential for both physical and cognitive health [1,2]. In particular, sleep plays an important role in sleep-dependent memory consolidation (SDMC). This process involves the reactivation and replay of temporary memories during sleep, which stabilizes them into long-term storage and facilitates their transfer from the hippocampus to the neocortex [3,4]. These features are thought to support memory consolidation; however, due to methodological challenges, SDMC remains underexamined in older adults, particularly those with subjective or mild cognitive impairment (MCI). Only a few studies have explicitly investigated SDMC in these populations, albeit in small sample sizes [5-8]. These scalability barriers have limited the field’s understanding of how factors such as modifiable dementia risk factors and global cognitive impairment influence SDMC.

Several factors may account for this gap in the literature, which currently constrains research on SDMC in populations at risk of dementia. For example, traditional SDMC assessment tasks were designed for younger or cognitively intact adults. Therefore, they are not suited for older adults with cognitive impairments, jeopardizing feasibility and producing floor effects. Furthermore, SDMC tasks have typically required assessment in the sleep laboratory before and after sleep [9-11]. The laboratory environment may not reflect naturalistic sleep conditions [12], limiting both the ecological validity and scalability of SDMC assessment for research and clinical use. While this setting offers a controlled environment, the requirement to stay overnight in the laboratory deems data collection resource-intensive, costly, and a limiting factor for larger-scale studies.

Digitalization of SDMC assessments offers an opportunity to address these limitations by enabling participants to complete memory tasks remotely and in their habitual sleeping environment. However, the feasibility of transiting from the laboratory to home assessments for SDMC tasks and willingness to participate in the research need to be tested. Both feasibility and willingness to participate may be influenced by demographics, clinical, and sleep-related factors as well as barriers such as technical issues, psychological concerns, and workload demands [13]. These considerations are especially important in memory clinic settings and specialist services that assess and support individuals with cognitive concerns, where digital tools are increasingly being considered to support assessment and intervention [14,15].

Recognizing the need for feasible SDMC tasks for older people with cognitive concerns, we initially developed a 32-item word-pairs task [5] in the sleep laboratory and demonstrated its use in older adults, including those with MCI. We then co-designed a mobile app named *Sleep Memories* [12,16] that delivers the 32-item task remotely, enabling at-home unsupervised assessment of SDMC. This research tool allows participants to complete the SDMC task at their convenience in their natural sleep environment, increases accessibility for those unable to attend the laboratory, and reduces research costs by eliminating the need for in-lab supervision. In this pilot study, the primary aim was to evaluate *willingness to participate* and the *feasibility* of implementing the *Sleep Memories* app in a memory clinic setting. Secondarily, we explored various demographic, clinical, and subjective sleep-related factors that may influence both willingness to participate and feasibility of the app. Furthermore, a subset of participants was asked for *user experiences and barriers* related to using the app for assessing the *acceptability of the app* in a memory clinic setting. Next, we explored the associations of demographics, clinical, subjective sleep-related factors, modifiable dementia risk factors, and global cognitive functioning with SDMC performance. Finally, we further explored differences in characteristics between low and high SDMC performers and compared the characteristics of participants who provided user feedback with the main sample. Feasibility was defined, in accordance with prior research, as the completion rate above 50% [17] and was hypothesized to be achievable in our study. We also hypothesized that willingness to participate would depend on demographics, clinical, and subjective sleep-related factors; specifically, it would be negatively associated with higher levels of cognitive impairment and lower global cognitive functioning, as well as poorer sleep behaviors.

## Methods

### Participants Recruitment

Between April and December 2024, we approached all consecutive participants attending the Healthy Brain Ageing clinic at the Brain and Mind Centre, University of Sydney, for participation. This clinic is a specialist memory and cognition clinic for older adults (aged 50 y or older) with new-onset cognitive or mood concerns. As previously described [18], the exclusion criteria for the clinic were: insufficient English to complete neuropsychological assessment, Mini-Mental State Examination (MMSE) score <20 [19], current substance dependence, intellectual disability, presence of nonaffective psychiatric disorder (eg, attention-deficit/hyperactivity disorder and schizophrenia), presence of neurological disease (eg, Parkinson and epilepsy), history of head injury with loss of consciousness >30 minutes, and history of stroke. Additional exclusion criteria for this study were the absence of a mobile phone and a diagnosis of dementia. Four participants were excluded for not having a mobile phone.

### Procedure

#### Overview

All participants underwent comprehensive and standardized neuropsychological, medical, and mood assessments conducted by a neuropsychologist, a geriatrician or neurologist, and a research psychologist, respectively. The *Sleep Memories* app was introduced to participants during their mood assessment. Those who expressed interest and provided informed consent were assisted by a researcher in installing the app on their mobile phones. Participants were also given written instructions on its use and subsequently completed memory tasks at-home in the evening and the following morning. Additionally, participants received text reminders to complete the task in the evening. Given that the sleep benefits for sleep-dependent consolidation of verbal memory are well established [3,4], and as this study was designed to assess feasibility rather than to test sleep-dependent memory effects, we did not include a control condition (eg, a wake retention interval).

#### Neuropsychological Assessment

A standardized battery of tests was administered by a neuropsychologist, covering a range of domains including premorbid IQ, attention, processing speed, working memory, verbal learning and memory, language, visuospatial and executive functions, and general cognitive functioning. The neuropsychological scores were adjusted for each participant’s age and level of education using normative data. For this study, we reported only the premorbid IQ, as assessed by the Wechsler Test of Adult Reading [20], global cognition, as assessed by the MMSE [19], and verbal learning (trials 1‐5) and memory (trial 7), as assessed by the Rey Auditory Verbal Learning Test [21].

Participants who were referred to the clinic by their general practitioner for the assessment of new-onset cognitive or mood concerns were classified as either subjective cognitive decline (SCD) or MCI based on standardized criteria through a consensus meeting involving a medical specialist and 2 neuropsychologists. Participants were classified as having MCI when they scored at least 1.50 SDs below normative values for their age and education on one or more neuropsychological tests, while overall functional independence remained intact. Participants meeting criteria for MCI were further subtyped based on the domain of cognitive impairment [22]. Those with deficits primarily in the memory domain were classified as having amnestic MCI, while those with impairments in nonmemory domains (eg, language, executive function, and visuospatial skills) were classified as having nonamnestic MCI. Participants were classified as having SCD if they self-reported a persistent decline in cognitive capacity compared to a previously normal status, in the absence of objective cognitive impairment on standardized neuropsychological testing. The decline was not caused by an acute event, and was not explained by MCI, prodromal Alzheimer disease, or other psychiatric, neurological, medical, or substance-related causes [23,24].

#### Medical Assessment

A medical assessment was conducted by a geriatrician or a neurologist who recorded the medical history and rated the medical burden using the Cumulative Illness Rating Scale-Geriatric [25]. Each system is scored from 0 to 4, and the total score of Cumulative Illness Rating Scale-Geriatric ranges from 0 to 52, with higher scores indicating greater illness severity and comorbidity.

#### Mood Assessment

A research psychologist assessed lifetime and current mood and anxiety symptoms using the Mini International Neuropsychiatric Interview [26]. They also used the Cogsleep Semi-structured Interview Revised Version [27] to probe for various sleep concerns. For this study, we reported the data on bedtime, wake-up time, and frequency (days) of trouble waking up in the morning from the semistructured interview, as individual sleep patterns and morning alertness might influence willingness to participate and task completion [28]. In addition, self-reported sleep quality was assessed using the Pittsburgh Sleep Quality Index [29], with higher global scores indicating poorer sleep quality. Insomnia symptoms were assessed using the Insomnia Severity Index [30], with higher scores indicating greater severity of insomnia symptoms. For depression and anxiety symptoms, the Geriatric Depression Scale-15 [31] and Geriatric Anxiety Inventory [32] were administered, respectively, with higher scores indicating greater levels of depression and anxiety.

### Sleep Memories App

#### Development

As previously described [12,16], the *Sleep Memories* mobile app was designed to deliver the 32-item word-pairs task via a chatbot named “Aurora.” Aurora used a low-cost internal chatbot engine that ran on the user’s device and required no Internet access. The engine was developed at Commonwealth Scientific and Industrial Research Organisation (CSIRO) and has been used extensively in other health applications [33,34] requiring ethical and safe operation. The app was co-designed by the authors and Healthy Brain Ageing researchers who provided the word-pairs task list and conducted focus groups and CSIRO software engineers who created and tested the chatbot architecture. They gathered inputs on the app’s functionality and accessibility from CSIRO staff (who tested it on their devices) [16] and older adults (4/11 were male, mean age 68.50 [SD 5.10] years; 8 had SCD and 3 had MCI) from the Healthy Brain Ageing clinic (focus group discussion) [12].

#### Evening Procedure

**Figure 1.**
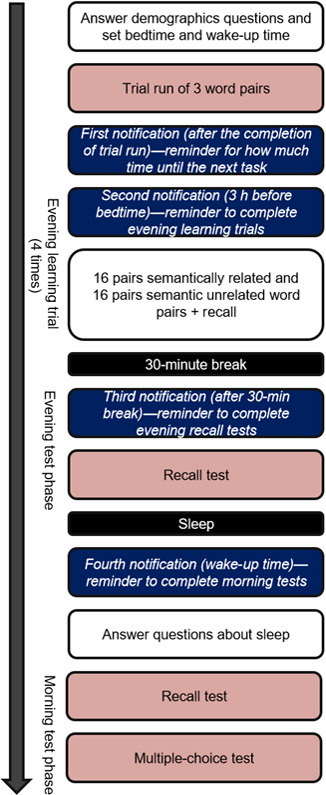
*Sleep Memories* app task flow. The figure was modified from the past study [5].

Figure 1 shows the time course and details of the app setup and completion. Upon starting the app, the chatbot collected basic demographic information, including age, gender, sleep time, and wake-up time from participants. Participants were then provided with a trial demonstration run of 3 word pairs to become familiar with the task and the app’s functionality. Participants were verbally instructed to complete the following tasks at home in a quiet, distraction-free environment and to refrain from using external aids.

After the demonstration, the chatbot informed participants to begin the word-pairs task within the 3-hour period preceding their reported sleep time. At the scheduled time, the participants received a notification to begin. They initially completed 4 learning trials. Each learning trial entailed a random presentation of 16 semantically related pairs (eg, syrup-maple) and 16 semantically unrelated word pairs (eg, groom-minimum). Each pair was presented for 10 seconds. At the end of each trial (ie, after all the word pairs had been presented), the participants were presented with the first word of each pair (eg, syrup) and were asked to provide the matching word (eg, maple) of each pair either by text or an audio response. The participants had 15 seconds to respond for each word pair. This was repeated for all 4 trials.

Following a 30-minute break, the participants were asked again to recall the word pairs during the evening recall test. The first word of each pair was presented for 15 seconds, and participants were asked to recall the matched word that had been paired with it. The entire evening session, excluding the break, lasted approximately 45 minutes. Participants were then able to sleep as usual.

#### Morning Procedure

**Figure 2.**
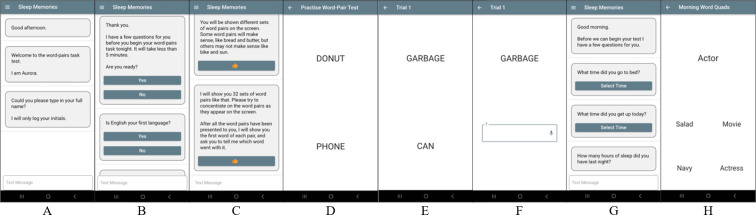
Screenshots of the *Sleep Memories* app. (A) The Aurora chatbot introducing themselves to the participant. (B) The app probing for demographic questions, sleep time, and wake time before learning trials. (C) The app providing instructions for the word-pairs task. (D) Trial run of the word-pairs task. (E) Learning component of the word-pairs task. (F) Recall component of the word-pairs task. (G) The app requesting details of the participants’ sleep before the morning tasks. (H) Multiple-choice testing component of the word pairs. The figure was modified from the past study [5].

Approximately within an hour after sleep offset, based on the wake-up time set in the app, the chatbot prompted participants to answer a few questions about their sleep and to confirm their sleep time and wake time. This was followed by two tests: (1) a morning delayed recall test whereby the first word of each pair was shown, and participants had to type or verbalize the matching word pair; and (2) a morning multiple-choice recognition test, whereby the first word of each pair was shown, along with 1 correct answer and 3 incorrect answers, and participants were asked to choose the correct matching word pair. To encourage task completion, the *Sleep Memories* app sent 4 notifications reminding participants to complete the tasks (see Figure 2 for screenshots of the *Sleep Memories* app). *Sleep Memories* logged all data on a Google Firebase service, with the server located within Australia.

Participants reported their estimated sleep-onset and wake-up times when setting up the app in the evening of testing, and these were confirmed again upon waking. In addition, the app automatically recorded the timing of the encoding and recall sessions, allowing us to derive the intervals between them.

At the data analysis stage, the SDMC score (% retention) was calculated by dividing the morning delayed recall test score by the evening recall test score and then multiplying by 100 (the evening recall test measured memory retention 30 min after learning the word pairs; the morning delayed recall test assessed memory retention after a night of sleep, approximately within an hour after waking).

#### Follow-Up

Within 2 weeks of task completion, a subset of participants was contacted to provide feedback on their user experiences (they were selected based on availability and successful contact). Researchers conducted phone interviews using a semistructured questionnaire as a guide (see Multimedia Appendix 1). During these interviews, the researchers summarized participants’ verbal responses, rather than recording or transcribing their verbatim as we did not have ethical approval for voice recording. Thus, verbatim transcription was not available. Importantly, the researchers involved in the questionnaire development and the qualitative analysis were independent of the *Sleep Memories* app development.

#### Feasibility and Acceptability of the *Sleep Memories* App

The feasibility of the *Sleep Memories* app was operationally defined as a completion rate of over 50% across all tasks, including evening learning trials, evening recall test, morning delayed recall test, morning multiple-choice test, and both evening recall and morning delayed recall tests [17]. This threshold was set based on prior studies of app-based cognitive assessments in older populations [35-37], reflecting the challenges of unsupervised digital cognitive assessment in older adults. It aligns with the Pragmatic-Explanatory Continuum Indicator Summary framework, which emphasizes pragmatic feasibility over idealized adherence [38]. While a scoping review of mobile-based cognitive assessments in healthy older adults reported completion rates around 60% [39], we set a slightly lower threshold of 50% as our sample included older adults with cognitive concerns who may face greater barriers to engagement with digital cognitive assessment [40].

The acceptability of the app was assessed qualitatively using a semistructured questionnaire (see Multimedia Appendix 1) developed through a bottom-up approach based on findings from prior focus group research [12]. While the Theoretical Framework of Acceptability [41] was not used as a blueprint during the initial design phase, the questionnaire sections align with the theory. Specifically, it captured: Intervention Coherence (sections 1 and 2 on app setup, layout, and clarity of instructions), Self-efficacy (sections 3 and 4 on the SDMC task and feasibility), Affective Attitude (section 5 on subjective feelings toward the app), and Burden (any reported task demands and obstacles). Furthermore, the questionnaire was reviewed by an experienced neuropsychologist and a researcher specializing in qualitative methodologies.

### Modifiable Dementia Risk Factors

The Lifestyle for Brain Health (LIBRA) index is a composite measure of 12 modifiable dementia risk factors, developed through a systematic review and expert consensus using the Delphi method [42]. The LIBRA 2 index is an updated version that incorporates additional factors, including hearing impairment, social contact, and sleep disturbances [42]. In this study, we used the validated LIBRA2 index to calculate a composite risk score by summing the available factor scores for each participant, with a theoretical range of 0 to 100. Positive weights were assigned when risk factors were present or protective factors were absent [42] (Table 1), with higher scores indicating greater dementia risk. In our sample, data were available for 13 of the 15 LIBRA2 factors, including high physical activity, sleep disturbances, high alcohol consumption, hearing impairment, kidney disease, coronary heart disease, diabetes, high cholesterol, smoking, hypertension, obesity, depression, and high cognitive activity. Data for healthy diet and low social contact were not available. Following previously published methodology [43], when less than 4 factors were missing, scores were calculated using the available factors. Four participants had 4 or more factors missing; therefore, these participants were excluded from LIBRA 2 analyses.

**Table 1.** Weights of the modifiable dementia risk factors included in Lifestyle for Brain Health (LIBRA) 2 Index^a^.

Factors	Type of factors	Measurements	Weight assigned
High physical activity	Protective	At least 150 minutes of activities and at least 5 sessions of activity over 1 week	6
Sleep disturbances	Risk	PSQI^b^ global score ≥5	3.3
High alcohol consumption	Risk	AUDIT-C^c^ total score (≥3 for women and ≥4 for men)	3.1
Hearing impairment	Risk	Self-report (yes)	7.6
Kidney disease	Risk	Self-report (yes)	5.7
Coronary heart disease	Risk	Self-report (yes)	8.3
Diabetes	Risk	Self-report (yes)	6.8
High cholesterol	Risk	Self-report (yes)	8.2
Smoking	Risk	Self-report (current smoking)	7.9
Hypertension	Risk	Self-report (yes)	3.5
Obesity	Risk	BMI ≥30	7
Depression	Risk	GDS-15^d^ total score ≥6	13
High cognitive activity	Protective	Highest tertile of CSA^e^ total score	9.4
Healthy diet	Protective	—^f^	—
Low social contact	Risk	—	—

a Positive weights were allocated whenever the risk factors were present, or the protective factors were absent.

b PSQI: Pittsburgh Sleep Quality Index.

c AUDIT-C: Alcohol Use Disorders Identification Test.

d GDS-15: Geriatric Depression Scale-15.

e CSA: Cognitively Stimulating Activities Questionnaire.

f Not available.

### Participant Group Definitions

We categorized participants into several groups. First, those who agreed versus those who declined to use the app. Second, those who completed all recall trials (full completers) versus those who did not complete 1 or both of the following: evening recall test and morning delayed recall test (partial completers). For additional exploratory data analysis, the median score for SDMC % retention was 92.40% (IQR 84.31-100.00). From this, we categorized participants as either low (n=18, <92.40) or high (n=18, ≥92.40) SDMC performers.

### Ethical Considerations

This study was approved by the University of Sydney Human Ethics Committee (2019/HE000271) and conducted in accordance with the World Medical Association Declaration of Helsinki. All participants provided informed consent before participating and were given the option to withdraw from the study at any time. The collected data were deidentified to ensure participant privacy and confidentiality. No compensation was provided to participants.

### Data Analysis

We conducted both quantitative data analysis and summarized user feedback on the *Sleep Memories* app. Quantitative data analysis was conducted using IBM SPSS Statistics (version 29) [44]. All continuous variables were inspected for normality using visual inspection (histograms), while the assumption of homogeneity of variance for these data was checked using the Levene test for equality of variances. For group comparisons, independent samples *t* tests were used for continuous variables that met assumptions of normality and homogeneity of variance. When the assumption of equal variances was violated, the Welch *t* test was applied. For nonnormally distributed variables, the nonparametric Mann-Whitney *U* test was used. Categorical variables were compared using chi-square tests. Prior to examining associations between SDMC performance and other variables (demographics, clinical factors, sleep behaviors, modifiable dementia risk factors, and global cognitive functioning), linear relationships were assessed visually using scatterplots. Following these assessments, the relationships between variables were analyzed using Pearson *r* for normally distributed variables and Spearman ρ for nonnormally distributed variables. We then compared the demographics, clinical factors, and subjective sleep-related factors between high and low SDMC performers as well as between participants who provided user feedback and those in the main sample.

Next, we summarized user feedback on the app. This analysis was based on open-ended questions at the end of each questionnaire section (see Multimedia Appendix 1), allowing participants to describe their experiences, usability, and challenges. The Likert-scale items were used only to guide discussion and provide context; no formal statistical analysis was conducted due to the small sample size (n=22), and participants’ responses were optional. We reported only the descriptive summary of selected items. The data analysis was conducted in an unbiased manner, with independent coding and theme identification and development carried out by 2 researchers (CCYT and SS). Following this, a meeting was held to compare and discuss their interpretations. Any discrepancies in coding or pattern identification and development were resolved through discussion, with a third researcher, AL, facilitating consensus when needed. To ensure impartiality, CCYT and SS were blinded to participants’ age, clinical diagnosis, and other personal information. Researchers identified patterns first; only then did they examine participants’ age, clinical diagnosis, and task completion to assess specific difficulties completing the tasks. The summarization of user feedback was conducted based on a four-step procedure: (1) becoming familiar with the data, (2) generating initial codes, (3) clearly defining and naming them, and finally, (4) producing writing that presents a coherent narrative supported by data extracts [45].

## Results

### Participants and Willingness to Participate

**Figure 3.**
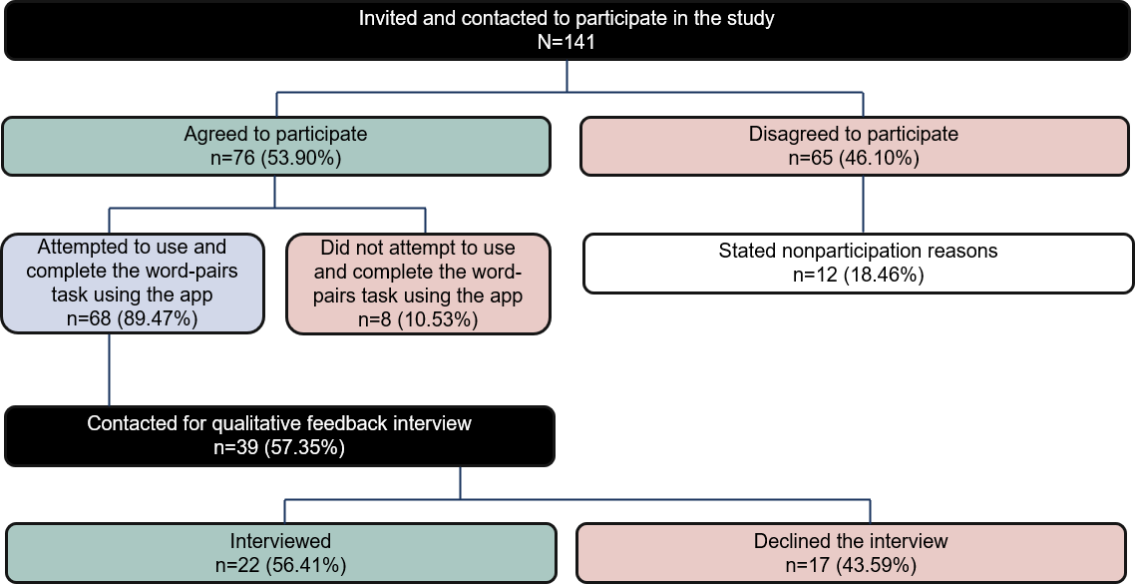
Recruitment and completion summary.

A total of 141 consecutive clinic attendees during the data collection period were invited to participate. Of these, 54% (n=76) agreed to participate. Of those who agreed, 89% (n=68) attempted the word-pairs task using the app. A subset (n=22) was contacted for postuse feedback (see Figure 3, which details the recruitment process, including numbers who agreed or declined, task completion, and participation in interviews). Additionally, 11 participants provided explicit reasons for nonparticipation during their clinic visit, including task too lengthy (n=1), lack of time (n=2), feeling overwhelmed by the task (n=2), difficulty downloading the app (n=2), struggling with technology (n=1), inability of the phone to scan the QR code (n=1), perceived lack of memory benefits (n=1), and forgetting to bring their phone (n=1).

### Feasibility of the *Sleep Memories* App

Of the 68 participants who attempted to use the *Sleep Memories* app to complete the word-pairs task, 68% (n=46) completed the evening recall test, 69% (n=47) completed the morning delayed recall test, and 57% (n=39) completed both tests. Out of the 46 participants who completed the evening recall test, 15% (n=7) completed only the evening recall test and not the morning delayed recall test. Similarly, of the 47 participants who completed the morning delayed recall test, 17% (n=8) completed only the morning delayed recall test and not the evening recall test.

The feasibility of the *Sleep Memories* app was assessed by examining the completion rate of the tasks: evening learning trials 1 to 4 (58/68, 85%; 57/68, 84%; 60/68, 88%; 60/68, 88%; respectively), evening recall test (46/48, 68%), morning delayed recall test (47/68, 69%), morning multiple-choice test (45/68, 66%), and both evening and morning recall tests (39/68, 57%)—all tasks had completion rates above 50% (Table 2). As described in the *Data Analysis* section, after adjusting for outliers, we computed the mean SDMC score of 92.76 (SD 13.85). In addition, the chi-square test showed no significant differences in completion rates between SCD and MCI groups (Table 3).

**Table 2.** User uptake of the *Sleep Memories* app (N=68).

Type of task	Values, n (%)^a^	95% CI
Evening learning trial 1	58 (85.29)	74.15‐92.35
Evening learning trial 2	57 (83.82)	72.47‐91.27
Evening learning trial 3	60 (88.24)	77.59‐94.42
Evening learning trial 4	60 (88.24)	77.59‐94.42
Evening recall test	46 (67.65)	55.09‐78.20
Morning delayed recall test	47 (69.12)	56.60‐79.46
Morning multiple-choice test	45 (66.18)	53.59‐76.93
Completed both evening recall and morning delayed recall tests	39 (57.35)	44.80‐69.07

a n (%) represents number of users completed and completion rate.

**Table 3.** User uptake of the *Sleep Memories* app in participants with subjective cognitive decline (SCD) versus mild cognitive impairment (MCI).

Type of task	*χ*^2^ (*df*)	*P* value	φ
Evening learning trial 1	0.61 (1)	.44	−0.09
Evening learning trial 2	0.13 (1)	.72	−0.04
Evening learning trial 3	0.05 (1)	.82	−0.03
Evening learning trial 4	0.05 (1)	.82	−0.03
Evening recall test	2.40 (1)	.12	0.19
Morning delayed recall test	0.12 (1)	.73	−0.04
Morning multiple-choice test	0.06 (1)	.81	−0.03
Completed both evening recall and morning delayed recall tests	0.001 (1)	.98	0.004

Among those who completed both evening recall and morning delayed recall tests, on average, the interval between evening encoding and sleep onset was 98.49 (SD 56.55) minutes. In the morning, on average, the interval between wake-up time and morning delayed recall was 33.61 (SD 37.85) minutes.

### Factors Influencing Willingness to Participate and Feasibility of the *Sleep Memories* App

#### Characteristics of Individuals Who Agreed and Disagreed to Use the *Sleep Memories* App

Participants who agreed to use the *Sleep Memories* app were more likely to have SCD than MCI, with higher MMSE scores, and tended to experience less difficulty waking up in the morning compared to those who declined participation (Table 4). There were no significant differences in other demographics, clinical factors, and sleep behaviors between those who agreed and those who disagreed to participate.

**Table 4.** Characteristics of participants who agreed to participate (n=76) and disagreed to participate (n=65).

Variable domains and variable	Agreed (n=76)	Disagreed (n=65)	Test statistic (*df*)	*P* value	Effect size
Demographics					
Age (y), mean (SD)	70.19 (7.75)	72.53 (7.08)	−1.86 (139)^a^	.07	0.31^b^
Education^c^ (y), mean (SD)	14.72 (2.77)	14.67 (3.46)	0.08 (131)^a^	.94	0.02^b^
Sex: females, n (%)	55 (72.37)	43 (66.15)	0.64 (1)^d^	.42	−0.07^e^
Vocational: working, n (%)	30 (39.47)	20 (30.77)	0.88 (1)^d^	.35	0.08^e^
Clinical factors					
Cognitive functioning^c^: MMSE^f^, mean (SD)^g^	28.95 (1.56)	27.90 (2.73)	1725.50^h^	.03^i^	0.03^j^
Depression^g^: GDS-15^k^, mean (SD)	2.95 (2.88)	3.34 (3.74)	2438.00^h^	.89^i^	0.00^j^
Anxiety^g^: GAI^l^, mean (SD)	4.01 (3.98)	4.83 (5.67)	2416.00^h^	.82^i^	0.00^j^
Clinical diagnosis: MCI^m^, n (%)	34 (44.74)	46 (70.77)	13.10 (1)^d^	<.001	−0.31^e^
Sleep-related factors, mean (SD)					
Sleep quality: PSQI^n^	6.47 (3.33)	6.88 (3.62)	−0.69 (139)^a^	.49	0.12^b^
Cogsleep^o^: bedtime	22:27 (1.02)	22:16 (1.34)	0.80 (139)^a^	.43	0.09^b^
Cogsleep^o^: wake time	6:27 (1.09)	6:51 (1.38)	−1.63 (112.75)^a^	.11	0.20^b^
Cogsleep^o^: trouble with waking up in the morning, number of days^c^	3.67 (2.40)	5.04 (2.25)	−2.04 (46)^a^	.047	0.59^b^
Insomnia^g^: ISI^g^^p^	7.17 (5.36)	7.14 (6.18)	2353.00^h^	.63	0.002^j^

a *t* test.

b Hedges *g*.

c Missing data for some participants.

d *χ*2 test.

e Phi.

f MMSE: Mini-Mental State Examination.

g Mann-Whitney *U* test was conducted for these nonnormally distributed variables. The assumptions of Mann-Whitney *U* test, including dependent variables at a continuous level, an independent variable consisting of 2 independent groups, and similarly shaped distributions between groups, were assumed. The test compared medians between the groups since the distributions between groups had the same shape.

h Mann-Whitney *U* test.

i *P* value for Mann-Whitney *U* test, the asymptotic significance was reported for ≥20 per group.

j Eta-squared.

k GDS-15: Geriatric Depression Scale-15.

l GAI: Geriatric Anxiety Inventory.

m MCI: mild cognitive impairment.

n PSQI: Pittsburgh Sleep Quality Index.

o Cogsleep: Cogsleep Semi-structured Interview Revised Version.

p ISI: Insomnia Severity Index.

#### Characteristics of Full Completers and Partial Completers

Full completers (completed both the evening recall and morning recall tests) had significantly more years of education and higher anxiety levels compared to partial completers (Table 5). There were no significant differences in other demographics, clinical factors, and sleep behaviors between these 2 groups.

**Table 5.** Characteristics of full completers (n=39) and partial completers (n=29).

Variable domains and variables	Full completer (n=39)	Partial completer (n=29)	Test statistic (*df*)	*P* value	Effect size
Demographics					
Age (y), mean (SD)	69.87 (8.02)	71.61 (7.41)	−0.91 (66)^a^	.37	0.22^b^
Education (y), mean (SD)^c^	15.19 (2.65)	13.76 (2.85)	2.06 (62)^a^	.04	0.52^b^
Sex: females, n (%)	26 (86.67)	23 (79.31)	1.32 (1)^d^	.25	0.14^e^
Vocational: working, n (%)	17 (43.59)	10 (34.48)	0.42 (1)^d^	.52	0.08^e^
Clinical factors					
Cognitive functioning^c^: MMSE^f^, mean (SD)^g^	29.03 (1.48)	28.79 (1.66)	466.00^h^	.28^i^	0.02^j^
Depression^g^: GDS-15^k^, mean (SD)	2.46 (2.52)	3.10 (2.60)	456.00^h^	.17^i^	0.03^j^
Anxiety^g^: GAI^l^, mean (SD)	4.64 (4.08)	2.69 (3.61)	387.50^h^	.03^i^	0.07^j^
Clinical diagnosis: MCI^m^, n (%)	16 (41.03)	12 (41.38)	0.001 (1)^d^	.98	−0.004^e^
Sleep-related factors, mean (SD)					
Sleep quality: PSQI^n^	6.21 (3.24)	6.62 (3.44)	−0.51 (66)^a^	.61	0.12^b^
Cogsleep^o^: bedtime	22:25 (0:59)	22:21 (1:09)	0.26 (66)^a^	.79	0.05^b^
Cogsleep^o^: wake time	6:20 (1:12)	6:36 (1:07)	−0.91 (66)^a^	.37	0.15^b^
Cogsleep^o^: trouble with waking up in the morning, number of days^c^	2.55 (2.24)	3.75 (2.19)	−1.14 (16)^a^	.27	0.54^b^
Insomnia^g^: ISI^p^	7.31 (4.85)	6.21 (5.46)	470.00^h^	.24	0.02^j^

a *t* test.

b Hedges *g*.

c Missing data for some participants.

d *χ*2 test.

e φ value.

f MMSE: Mini-Mental State Examination.

g Mann-Whitney *U* test was conducted for these nonnormally distributed variables. The assumptions of Mann-Whitney *U* test, including dependent variables at a continuous level, an independent variable consisting of 2 independent groups, and similarly shaped distributions between groups, were assumed. The test compared medians between the groups since the distributions between groups had the same shape.

h Mann-Whitney *U* test.

i *P* value for Mann-Whitney *U* test, the asymptotic significance was reported for ≥20 per group.

j Eta-squared.

k GDS-15: Geriatric Depression Scale-15.

l GAI: Geriatric Anxiety Inventory.

m MCI: mild cognitive impairment.

n PSQI: Pittsburgh Sleep Quality Index.

o Cogsleep: Cogsleep Semi-structured Interview Revised Version.

p ISI: Insomnia Severity Index.

### User Feedback and Acceptability of the *Sleep Memories* App in a Memory Clinic Setting

First, we present a brief descriptive summary of selected Likert-scale items from the Sleep Memories App Feedback Questionnaire (see Multimedia Appendix 1). Overall, 82% (18/22) of participants agreed that the app instructions were easy to understand and that the task was straightforward. A majority (20/22, 91%) indicated a preference for completing the task using the app rather than in a sleep laboratory. Additionally, 86% (19/22) reported an overall positive experience with the app. Notably, none of the participants reported needing help from their family to complete the SDMC task, while only 14% (3/22) reported needing family assistance to navigate the app.

Next, qualitative feedback from participants described age-related physical and cognitive changes that influenced their experience with the app. More specifically, 2 participants commented that the app appeared more user-friendly for individuals who are younger or more computer-literate, rather than for older users. One participant noted that being from a generation familiar with computers, but not mobile phones, made completing the task on an unfamiliar device challenging. Another suggested that improvements could be made to better accommodate individuals with vision or dexterity impairments.

Participants generally provided positive feedback, describing the app as easy to use, user-friendly, and well-designed, with some finding it fun, interesting, and convenient to complete tasks at home. One participant even shared their positive experiences with others, and another described it as one of the best apps they had ever used. Less positive feedback included feelings of nervousness or stress during testing, concerns about performance, and perceptions that the app was less suitable or engaging for older users. Given that the app is designed for cognitive testing, some level of anxiety is expected.

In addition, participants reported several technical challenges related to the functionality of the *Sleep Memories* app and the clarity of the accompanying instructions. Participants also raised concerns regarding the app’s accessibility and suitability for older adults and individuals with additional needs. The most frequently mentioned issue was tasks being delivered too quickly, making it difficult to keep up. Nevertheless, they appreciated the voice input feature, making task completion easier (see Table 6 for more details).

**Table 6.** Summary of user feedback.

Patterns and comments	Participants, n
Subjective experiences	
Positive	
Easy to use, user-friendly, and straightforward	3
Well set up	1
Good layout	1
Convenient as it allowed tasks to be completed at home	1
Simple tap-based multiple-choice test	1
Interesting process	1
Shared positive experience with others	1
Best apps they had ever used	1
Less positive	
Nervous or stressed while completing the tests	3
Worried about their performance	2
Not suitable for older users	2
The app was “dry”	1
Technical challenges	
Instruction sheet provided on how to use the app was unclear	1
Needed more explanation for the morning tasks	1
Misunderstood the evening task notification as requiring immediate completion	2
Unclear wording of the second morning task (multiple-choice)	2
Unawareness that questions would automatically skip after a time limit	3
Lack of instruction on what to do if they did not know an answer	1
Confusion over morning task timing based on wake-up time	1
Delays in morning task delivery	2
Suggested the need to visually distinguish cues from response options in the multiple-choice task	2
App’s accessibility	
Tasks being delivered too quickly, making it difficult to keep up	5
Challenges using the microphone	2
Difficulty with touch functions on mobile devices due to aging	1
Needed assistance from family members to navigate the app and difficulty using it independently	1
Experienced illness during testing	2
Questioned whether individuals with dementia might become frustrated using the app	1
Voice input feature was helpful and made task completion easier	1

### Predictors of SDMC Performance

There were no differences in SDMC across gender (*t*_34_=−0.65, *P*=.52, Hedges *g*=0.23) and clinical diagnostic groups (*t*_34_=0.55, *P*=.59, Hedges *g*=0.18). We also examined the associations between SDMC performance and several variables: demographics, clinical factors, sleep behaviors, modifiable dementia risk factors, and global cognitive functioning. We first examined these relationships using all available SDMC data points. As shown in Table 7, there was a significant and positive correlation between premorbid IQ (as measured by Wechsler Test of Adult Reading) and SDMC. Nevertheless, there were no statistically significant correlations between SDMC and other demographics, clinical factors, sleep behaviors, and modifiable dementia risk factors. Since this was an exploratory study with a small sample size, we did not correct for multiple comparisons.

**Table 7.** Predictors of sleep-dependent memory consolidation (SDMC).

Variable domains and variables	Effect size	*P* value
Demographic		
Age	0.14^a^	.41
Years of education	0.10^a^	.57
Clinical factors		
Depression: GDS-15^b^	0.001^a^	.10
Anxiety: GAI^c^	0.25^a^	.13
Chronic disease: CIRS-G^d^	−0.006^a^	.97
Sleep behaviors		
Sleep quality: PSQI^e^	−0.11^a^	.51
Insomnia: ISI^f^	−0.05^a^	.74
Modifiable dementia risk factors		
LIBRA^g^ 2 index	0.01^a^	.94
Global cognitive functioning		
WTAR^h^ (premorbid IQ)	0.38^a^	.02
MMSE^i^	0.11^j^	.51
RAVLT^k^ (7/5% retention)	0.05^a^	.79

a Pearson *r*.

b GDS-15: Geriatric Depression Scale-15.

c GAI: Geriatric Anxiety Inventory.

d CIRS-G: Cumulative Illness Rating Scale-Geriatric.

e PSQI: Pittsburgh Sleep Quality Index.

f ISI: Insomnia Severity Index.

g LIBRA: Lifestyle for Brain Health.

h WTAR: Wechsler Test of Adult Reading.

i MMSE: Mini-Mental State Examination.

j Spearman ρ.

k RAVLT: Rey Auditory Verbal Learning Test.

Next, we conducted a sensitivity analysis to assess whether potential outliers on the evening recall task might influence the results. We excluded 3 evening recall data points that were SD 1.50 below the mean. These data points were excluded for memory consolidation-specific analyses (SDMC% retention) but retained for all other analyses. The SD 1.50 cutoff was selected as a pragmatic adaptation of common outlier detection rule [46]. Results with this exclusion are presented in Table 8, and findings were consistent both with and without the exclusion.

**Table 8.** Predictors of sleep-dependent memory consolidation (SDMC) excluding evening recall scores 1.50 SD below the mean.

Variable domains and variables	Effect size	*P* value
Demographic		
Age	0.01^a^	.95
Years of education	0.17^a^	.34
Clinical factors		
Depression: GDS-15^b^	−0.03^a^	.88
Anxiety: GAI^c^	0.23^a^	.19
Chronic disease: CIRS-G^d^	−0.06^a^	.73
Sleep behaviors		
Sleep quality: PSQI^e^	−0.12^a^	.48
Insomnia: ISI^f^	0.01^a^	.95
Modifiable dementia risk factors		
LIBRA^g^ 2 index	−0.10^a^	.57
Global cognitive functioning		
WTAR^h^ (premorbid IQ)	0.36^a^	.04
MMSE^i^	0.05^j^	.74
RAVLT^k^ (7/5% retention)	0.03^a^	.88

a Pearson *r*.

b GDS-15: Geriatric Depression Scale-15.

c GAI: Geriatric Anxiety Inventory.

d CIRS-G: Cumulative Illness Rating Scale-Geriatric.

e PSQI: Pittsburgh Sleep Quality Index.

f ISI: Insomnia Severity Index.

g LIBRA: Lifestyle for Brain Health.

h WTAR: Wechsler Test of Adult Reading.

i MMSE: Mini-Mental State Examination.

j Spearman ρ.

k RAVLT: Rey Auditory Verbal Learning Test.

### Exploratory Analyses

In terms of comparing the characteristics between low and high SDMC performers (Table 9), low performers had significantly less education and lower anxiety levels compared to high performers. There were no other differences between these 2 groups.

**Table 9.** Characteristics of low (n=18) and high (n=18) performers of sleep-dependent memory consolidation (SDMC) task.

Variable domains and variables	Low performer (n=18)	High performer (n=18)	Test statistic (*df*)	*P* value	Effect size
Demographics					
Age (y), mean (SD)	70.83 (6.77)	68.95 (8.96)	0.71 (34)^a^	.48	0.24^b^
Education (y), mean (SD)^c^	14.11 (2.35)	16.25 (2.59)	−2.52 (32)^a^	.02	0.87^b^
Sex: females, n (%)	10 (55.56)	14 (77.78)	2.00 (1)^d^	.16	0.24^e^
Vocational: working, n (%)	9 (50)	8 (44.44)	0.11 (1)^d^	.74	0.06^e^
Clinical factors					
Cognitive functioning: MMSE^f^, mean (SD)^g^	28.94 (1.51)	29.17 (1.51)	147.50^h^	.65^i^	0.006^j^
Depression^g^: GDS-15^k^, mean (SD)	2.50 (2.94)	2.56 (2.28)	146.50^h^	.63^i^	0.01^j^
Anxiety^g^: GAI^l^, mean (SD)	3.39 (3.99)	6.11 (3.94)	88.50^h^	.02^i^	0.15^j^
Clinical diagnosis: MCI^m^, n (%)	8 (44.44)	7 (38.89)	0.11 (1)^d^	.74	0.06^e^
Sleep-related factors, mean (SD)					
Sleep quality: PSQI^n^	6.00 (3.01)	6.61 (3.62)	−0.55 (34)^a^	.59	0.19^b^
Cogsleep^o^: bedtime	22:38 (1:04)	22:09 (0:53)	1.42 (34)^a^	.17	0.35^b^
Cogsleep^o^: wake time	6:21 (1:01)	6:12 (1:20)	0.40 (34)^a^	.69	0.08^b^
Cogsleep^o^: trouble with waking up in morning, number of days^c^	1.00 (0.00)	3.25 (2.64)	−2.09 (5)^a^	.09	1.21^b^
Insomnia^g^: ISI^p^	7.00 (4.41)	8.11 (5.05)	144.00^h^	.58^i^	0.009^j^

a *t* test.

b Cohen *d*.

c Missing data for some participants.

d *χ*2 test.

e Phi.

f MMSE: Mini-Mental State Examination.

g Mann-Whitney *U* test was conducted for these nonnormally distributed variables. The assumptions of the Mann-Whitney *U* test, including dependent variables at a continuous level, an independent variable consisting of 2 independent groups, and similarly shaped distributions between groups, were assumed. The test compared medians between the groups since the distributions between groups had the same shape.

h Mann-Whitney *U* test.

i *P* values for the Mann-Whitney *U* test, the exact significance was reported for the small sample size.

j Eta-squared.

k GDS-15: Geriatric Depression Scale-15.

l GAI: Geriatric Anxiety Inventory.

m MCI: mild cognitive impairment.

n PSQI: Pittsburgh Sleep Quality Index.

o Cogsleep: Cogsleep Semi-structured Interview Revised Version.

p ISI: Insomnia Severity Index.

When comparing the characteristics of participants who provided user feedback (subsample) with those of the main sample, the subsample included a higher proportion of individuals with SCD than the main sample. Nevertheless, there was no difference between the groups in the proportion of full and partial completers. There were also no other differences between these 2 groups (Table 10).

We also reported task completion rates among participants who reported difficulty in using the app (Table 11).

**Table 10.** Characteristics of user feedback provider (n=22) compared to the main sample (n=119).

Variable domains and variables	User feedback provider (n=22)	Main sample (n=119)	Test statistic (*df*)	*P* value	Effect size
Demographics					
Age (y), mean (SD)	72.18 (6.45)	71.10 (7.71)	0.61 (139)^a^	.54	−0.14^b^
Education (y), mean (SD)^c^	14.39 (2.99)	14.76 (3.12)	−0.51 (131)^a^	.61	0.12^b^
Sex: females, n (%)	19 (86.36)	79 (66.39)	3.50 (1)^d^	.06	−0.16^e^
Vocational: working, n (%)	7 (31.82)	43 (36.13)	0.25 (1)^d^	.62	−0.04^e^
Clinical factors					
Cognitive functioning^c^: MMSE^f^, mean (SD)^g^	29.18 (1.22)	28.35 (2.33)	1002.50^h^	.16^i^	0.01^j^
Depression^g^: GDS-15^k^, mean (SD)	2.64 (2.70)	3.22 (3.40)	1236.50^h^	.68^i^	0.00^j^
Anxiety^g^: GAI^l^, mean (SD)	4.05 (3.87)	4.45 (5.00)	1241.00^h^	.70^i^	0.00^j^
Clinical diagnosis: MCI^m^, n (%)	7 (31.82)	73 (61.34)	7.62 (1)^d^	.006	−0.24^e^
Sleep-related factors, mean (SD)					
Sleep quality: PSQI^n^	6.95 (2.34)	6.61 (3.64)	0.58 (42.44)^a^	.56	−0.10^b^
Cogsleep^o^: bedtime	22:33 (0.59)	22:20 (1.21)	0.72 (139)^a^	.47	−0.11^b^
Cogsleep^o^: wake time	6:25 (1.15)	6:41 (1.26)	−0.82 (139)^a^	.41	0.13^b^
Cogsleep^o^: trouble with waking up in the morning, number of days^c^	3.25 (2.87)	4.49 (2.36)	−0.99 (46)^a^	.33	0.52^b^
Insomnia^g^: ISI^p^	7.09 (4.21)	7.17 (5.98)	1212.00^h^	.58	0.002^j^
Word-pairs task completion, n (%)					
Task completion: completers	12 (57.14)^q^	27 (57.45)^r^	0.001 (1)^d^	.98	−0.003^e^

a *t* test.

b Hedges *g*.

c Missing data for some participants.

d *χ*2 test.

e Phi.

f MMSE: Mini-Mental State Examination.

g Mann-Whitney *U* test was conducted for these nonnormally distributed variables. The assumptions of the Mann-Whitney *U* test, including dependent variables at a continuous level, an independent variable consisting of 2 independent groups, and similarly shaped distributions between groups, were assumed. The test compared medians between the groups since the distributions between groups had the same shape.

h Mann-Whitney *U* test.

i *P* values for the Mann-Whitney *U* test, the asymptotic significance was reported for ≥20 per group.

j Eta-squared.

k GDS-15: Geriatric Depression Scale-15.

l GAI: Geriatric Anxiety Inventory.

m MCI: mild cognitive impairment.

n PSQI: Pittsburgh Sleep Quality Index.

o Cogsleep: Cogsleep Semi-structured Interview. Revised Version.

p ISI: Insomnia Severity Index.

q n=21.

r n=47.

**Table 11.** Task completion rates among participants reporting difficulty.

ID	Age (y)	Clinical diagnosis	Completion of 4 evening learning trials	Completion of evening test	Completion of morning test	Completion of morning multiple choice task	Notes
1	73.46	MCI^a^	Yes (all 4)	No (no score)	No (no score)	No (no score)	Experienced app malfunction and believed their data were incomplete
2	72.05	SCD^b^	No (all 4)	No (no score)	No (no score)	No (no score)	Unable to complete the task
3	70.88	SCD	Yes (all 4)	Yes	Yes	Yes	The screen went blank due to inactivity (not touching the screen)
4	76.37	SCD	Yes (all 4)	Yes	Yes	Yes	Encountered several issues with the app and could not complete the final morning task
5	73.77	MCI	Yes (all 4)	No (no score)	No (no score)	No (no score)	Thought there were 3 trials, and as a result, lost all previous results
6	70.61	SCD	Yes (all 4)	Yes	Yes	Partial (score=1)	Unable to complete the morning task
7	64.5	SCD	Yes (all 4)	Yes	Yes	Yes	Encountered an issue during the first attempt and had to clear the app data and try again

a MCI: mild cognitive impairment.

b SCD: subjective cognitive decline.

## Discussion

### Principal Findings

This study aimed to evaluate the willingness to engage with and the feasibility of using the *Sleep Memories* app to assess SDMC in older adults with cognitive concerns and impairments within a brain health and memory clinic setting. It is important to note that this study focused on the feasibility of a digital delivery platform for the SDMC task rather than providing objective physiological sleep metrics, such as those obtained through actigraphy or polysomnography.

Overall, the app was deemed feasible (>50% completion) as determined a priori. Notably, our study involved greater logistical demands than prior app-based cognitive studies. Sleep-dependent memory protocols require tighter scheduling around sleep periods and may involve overnight laboratory stays in unfamiliar environments, which are not always feasible for older adults with cognitive impairment. Therefore, the 57% completion rate, while modest, indicates practical feasibility for real-world implementation. Willingness to participate was higher among those with SCD than MCI, those with higher global cognitive scores, and those with less difficulty waking up in the morning. Additionally, task completion was higher among those with more years of education and higher anxiety levels.

Interestingly, while similar completion rates in the SCD and MCI groups support the app’s feasibility, the higher initial refusal rate among individuals with MCI and lower cognitive scores suggests a barrier to participation. This aligns with prior work, which showed that, relative to those with more severe cognitive impairments, individuals with SCD are often motivated to participate in research studies due to concern about cognitive decline, despite normal objective performance [47]. Future research could adopt a dyadic approach by involving a care partner to assist with technical issues at home, or potentially offering on-call technical support, helping reduce barriers for those who might otherwise decline participation.

In this study, we recorded reasons for nonparticipation from a subset of participants, which suggested that nonparticipation was influenced by a combination of perceived task burden (eg, participant perceived the word-pairs task as lengthy), technical barriers (eg, phone unable to download the app), and motivational factors (eg, participant perceived the task as lacking memory benefit). These findings are in accordance with the Unified Theory of Acceptance and Use of Technology [48], where “effort expectancy” and “facilitating conditions” correspond to the perceived task burden and technical barriers observed in this study, and “performance expectancy” reflects participants’ motivation to engage with the app based on perceived cognitive benefits. Moreover, consistent with past research linking higher global cognitive functioning to greater health literacy [49], our results revealed that individuals agreeing to participate had more superior global cognition compared to those with cognitive decrements. This may be suggesting that participants with higher global cognitive functioning might be more motivated to engage in research perceived as supporting sleep and cognitive health. Difficulty with sleep also appears to affect willingness to participate in sleep-dependent memory tasks. Waking difficulties often linked to disrupted sleep or fatigue. This may reduce motivation or capacity to engage in research activities in the morning, especially those requiring structured participation such as completing tasks via a mobile app with strict timings (ie, 3 h before sleep and upon waking). Analyses of other sleep-related apps reveal similar issues. For instance, “SleepFit,” which aims to enhance sleep hygiene among paramedics [50], had a substantial dropout rate of 91.40%. The authors attributed this low engagement and retention to fatigue and disrupted sleep, particularly making it difficult for users to adhere to the app’s scheduled tasks.

The *Sleep Memories* app demonstrated completion rates comparable to other remote cognitive assessments in older populations. For example, a study of older adults at risk of cognitive decline and dementia (mean age 57.3, SD 5.3 y), using the smartphone-based iVitality app (featuring tests adapted from conventional neuropsychological assessments) reported completion rates of approximately 60% [36]. Similarly, an unsupervised app−based study in healthy older adults, assessing memory recall and object and scene discrimination, reported completion rates ranging from 54% to 69% [35]. Notably, the completion rate observed in this pilot study exceeded that reported for the Linus Health Core Cognitive Evaluation app in older adults (mean age 74.0, SD 6.0 y), which achieved a substantially lower rate of around 10% [37]. In this study, we found a strong initial task completion rate, particularly during the learning phase. However, the task completion rate declined during the evening recall and morning recall tests, suggesting that the completion of delayed recall tasks administered either 30 minutes later (evening recall task) or the following morning (morning delayed recall and multiple-choice tasks) may pose greater challenges in remote, unsupervised contexts. Re-engagement with the app after a delay may be influenced by contextual factors such as interruptions, competing demands, or diminished motivation to return to the task [51,52]. Notably, completion rates for all learning trials, evening, and morning tasks did not differ significantly between participants with SCD and MCI. This finding is consistent with a past study that similarly showed no significant differences in online cognitive test completion based on cognitive status [53]. Additionally, based on user feedback provided, aging—especially physical and cognitive changes—affects task completion. For instance, for some, a decline in fine motor skills appeared to hinder typing accurately on a mobile screen, especially on smaller devices. Slowed reaction times and decreased hand-eye coordination further contributed to difficulties with tasks requiring precise tapping. Additionally, reduced visual acuity common in later life [54] might also impact the ability to read small on-screen instructions and navigate through mobile apps. These sensory and motor limitations are often compounded by a general age-related unfamiliarity with digital interfaces, resulting in reduced confidence in using mobile apps. Importantly, our results showed no differences in age between full completers and partial completers, suggesting that it is not chronological age itself but the effects of aging such as changes in motor, visual, and cognitive abilities that influence task completion. Other than physical and cognitive changes due to aging, education and psychological factors also play a role in affecting the completion of app-based SDMC tasks, as we found full completers tended to have more years of education and higher anxiety levels compared to partial completers. Education fosters cognitive discipline and familiarity with structured tasks [55,56], potentially making these individuals more likely to persist through challenges. Meanwhile, elevated anxiety (when managed effectively) can enhance task vigilance, increase motivation to avoid failure, and drive individuals to complete tasks with greater urgency [57]. A similar pattern was observed when we compared the characteristics of low and high performers of SDMC tasks—high performers had more years of education and higher anxiety levels than low performers. Together, these findings suggest that education equips individuals with strategies to manage tasks, and that anxiety can provide motivation for completion and performance in SDMC tasks. When examining user feedback from full completer and partial completer groups, full completers generally reported more positive subjective experiences, noting that the app was easy to use, enjoyable, and fun. Two participants from the full completer group mentioned feeling nervous during the task, possibly due to the evaluative or confrontational nature of cognitive testing and expressed concerns about their task performance. Partial completers reported similar experiences, indicating little difference between full completers and partial completers in terms of subjective experiences. In terms of technical challenges, some full completers reported that the instructional sheet was unclear and that the task progressed too quickly. It is important to note that substantially slowing down task delivery may risk introducing ceiling effects, making the task overly easy and unnecessarily lengthy. In contrast, partial completers provided little feedback regarding the app or technical difficulties. This lack of feedback may reflect lower motivation, earlier disengagement before issues arose, or a simple disinclination to comment. One participant in the partial completer group (diagnosed with MCI) reported needing assistance from family members to navigate the app. Although this was an isolated case, and the task had originally been designed and validated for people with MCI within the sleep laboratory [5], this observation suggests that caregiver support may be necessary to facilitate engagement with the *Sleep Memories* app among some users with greater cognitive impairment.

Having considered factors influencing willingness to participate and the feasibility of the app based on task completion, it is also important to understand those affecting SDMC performance. Notably, cognitive reserve affects SDMC performance. We observed a positive correlation between premorbid IQ and SDMC performance, which aligns with past research showing that cognitive reserve is linked to better cognitive outcomes [58]. Our findings suggest that premorbid IQ influences task performance and retention, underscoring the importance of considering cognitive reserve in remote assessments.

Given that the *Sleep Memories* app was used in an unsupervised, remote capacity, we acknowledge that contextual and behavioral factors may contribute to variability in SDMC scores. However, we found that except for premorbid IQ, none of the demographic, clinical, sleep behavior, modifiable dementia risk factors, or cognitive variables were significantly associated with overnight memory consolidation. This suggests that while such factors may introduce variability, they do not systematically account for individual differences in SDMC performance.

### Research Impact

This was the first study to demonstrate the feasibility of SDMC data collection delivered unsupervised through a chatbot and mobile app. While not a replacement for laboratory studies, the *Sleep Memories* app demonstrates potential as a complementary research tool that may support more accessible and cost-effective data collection.

The comparable task completion rates between participants with SCD and those with MCI indicate that the *Sleep Memories* app and the word-pairs task can be feasibly administered across these populations. Nevertheless, as with other digital cognitive tools, unsupervised SDMC testing presents challenges related to cognitive load and user engagement. In this study, we also highlighted key factors that may facilitate the adoption of the app, which could inform the development of personalized interventions aimed at optimizing healthy brain aging in the future.

Participant feedback emphasized the value of clear, concise instructions, and well-defined expectations regarding task timing and completion. To support usability among older adults, accessible design strategies are essential, such as larger icons, high-contrast text, intuitive audio cues, and customizable interface settings. Ultimately, refining and validating remote memory tools such as the *Sleep Memories* app across global cognitive and demographic profiles will be critical for advancing equitable, scalable cognitive health monitoring in clinical and community settings.

### Limitations

There are several limitations to this study. First, we did not record participants’ feedback. Future qualitative studies should consider audio-recording responses and conducting formal thematic analyses of verbatim data. Nonetheless, we identified several factors that influenced participation and completion rates. Second, we did not systematically assess the digital literacy of participants, which may have influenced their ability to use the app and interpret results. Third, as widely documented in the literature, the validity of remote cognitive testing must be carefully considered, given the potential for uncontrolled distractions and external assistance. Reassuringly, the observed scores in this study were comparable to those reported in similar lab-based SDMC studies involving comparable cohorts [5]. Nonetheless, incorporating additional validity safeguards and conducting larger-scale evaluations will be important to further mitigate these limitations. Fourth, the study did not directly validate against supervised lab-based SDMC assessments. However, complete equivalence should not be expected, given differences in delivery format and response conditions, and the observed scores were comparable to those reported in similar lab-based SDMC research in older and MCI samples [5]. Finally, sleep and wake times were self-reported, and no objective measures were collected to verify these estimates. Notwithstanding these limitations, the study has strengths worth mentioning. For instance, it demonstrates high ecological validity and that recruitment and SDMC task delivery via the *Sleep Memories* app are feasible for a memory clinic setting.

### Conclusions

This study demonstrated the feasibility of using the *Sleep Memories* app to assess SDMC in older adults within a memory clinic context. These findings offer valuable guidance for the development of accessible digital tools that can effectively monitor cognitive health across varying levels of impairment.

The team is currently continuing to collect SDMC data using the app from a larger sample recruited through a brain health and memory clinic setting to support ongoing refinement and larger-scale validation studies. These efforts aim to further establish the feasibility, reliability, and clinical utility of the app, the characterization of neurobiological correlates, and sensitivity to interventions and to inform scalability for community-based implementation.

## Supplementary material

10.2196/87926 Multimedia Appendix 1 Sleep Memories App Qualitative Feedback Questionnaire.

## Abbreviations

CIRS-G: Cumulative Illness Rating Scale-Geriatric
CSIRO: Commonwealth Scientific and Industrial Research
LIBRA: Lifestyle for Brain Health
MCI: mild cognitive impairment
MMSE: Mini-Mental State Examination
SCD: subjective cognitive decline
SDMC: sleep-dependent memory consolidation
