# Supplementary material for: Feasibility and Acceptability of a Remote Sleep–Dependent Memory Assessment in Older Adults With Cognitive Concerns: Pilot Cross-Sectional Study
Source: JMIR Aging. 2026 Jun 11;9:e87926. doi: 10.2196/87926 (PMC13256488; doi:10.2196/87926)
Supplement: Multimedia Appendix 1 [file aging-v9-e87926-s001.docx]

## Multimedia Appendix 1: Sleep Memories App Qualitative Feedback Questionnaire

Question 1: Preliminary feedback from subjects (general thoughts)

For the following section, the participants will rate from “Strongly Agree” to “Strongly Disagree”.

**Section 1:  Setting up the app, and navigation and layout.**

1. I did not need assistance downloading and setting up the app.
2. I did not need or receive help using the app.
3. I was able to navigate the app without any assistance.
4. I was confident navigating the app.
5. The app’s design and layout were intuitive and logical.
6. Text size was easy to read.
7. I did not find the app’s interface/ layout to be confusing.
8. Selecting time to go to bed and to wake up was easy.
9. Entering text into the text box was easy.

Did you have any suggestions for this category? Feel free to provide any other recommendations, concerns or ideas.

**Section 2: Instructions**

1. Chatbot instructions were easy to understand.
2. The chatbot provided simple straightforward commands.
3. I understood all Chatbot’s instructions.
4. I was able to follow all Chatbot commands.
5. The Chatbot’s voice felt natural.

Did you have any suggestions for this category? Feel free to provide any other recommendations, concerns or ideas.

**Section 3: Sleep-dependent memory consolidation task**

1. The task was straight forward and simple to understand.
2. I did not need or receive help completing the task.
3. The task was easy to complete.
4. I understood what I needed to do in the task.
5. The requirements of the task was straightforward.
6. The task felt appropriate for my age demographic.

 Did you have any suggestions for this category? Feel free to provide any other recommendations, concerns or ideas.

**Section 4: Feasibility**

1. I remembered to complete the task at the required times.
2. The app did not significantly disrupt my daily routine.
3. I didn’t need to be reminded when to take the task.
4. I did not struggle to remember to complete the task before sleeping.
5. I did not struggle to remember to complete the task in the morning.
6. I found the notifications to be helpful.
7. I did not find myself distracted with other unrelated activities when completing the task.
8. I would rather take the task on an app rather than attend a sleep study experiment conducted in a laboratory.

 Did you have any suggestions for this category? Feel free to provide any other recommendations, concerns or ideas.

**Section 5: Subjective feelings towards the app**

1. I feel as though the app has been designed in a user-friendly way.
2. I feel that the app takes into consideration the physical needs of elderly people e.g., motor dexterity, vision impairments etc.
3. I feel that the app takes into consideration the cognitive needs of elderly people e.g., attention and memory.
4. My overall experience with the app was positive.
5. I felt comfortable using the app.
6. I feel that the app is appropriate to use for my age demographic.

Did you have any suggestions for this category? Feel free to provide any other recommendations, concerns or ideas.
